# Perks of Rehabilitation in a Patient Who Underwent Total Hip Replacement Surgery Twice: A Case Report

**DOI:** 10.7759/cureus.46137

**Published:** 2023-09-28

**Authors:** Nandini C Baheti, Pallavi Harjpal

**Affiliations:** 1 Department of Physiotherapy, Ravi Nair Physiotherapy College, Datta Meghe Institute of Higher Education and Research, Wardha, IND; 2 Department of Neuro-Physiotherapy, Ravi Nair Physiotherapy College, Datta Meghe Institute of Higher Education and Research, Wardha, IND

**Keywords:** coxafemoral joint, postural stability, rehabilitation, physical therapy, total hip arthroplasty

## Abstract

The hip joint is called as the coxafemoral or femoroacetabular joint, and it is the articulation of the acetabulum of the pelvis and the head of the femur. The most common surgery in adults is total hip arthroplasty (THA). In this technique, biocompatible materials are used to replace sections of the upper femur and acetabulum. A postoperative patient needs physical rehabilitation and it is necessary to focus on strength and functional status. Instability appears to be a complication in both initial and repeat THA. A 56-year-old male met with an accident, and on consulting with an orthopedic surgeon, an X-ray scan was taken and was then advised for total hip replacement, post-surgery, he developed an infection and was again advised for hip replacement. Post-surgery, the patient received physiotherapy. The four elements of the physiotherapy rehabilitation regimen are therapeutic physical activity, transfers, gait training, and education in daily living skills. Following surgery, physiotherapy rehabilitation may be provided at various times, including right away afterward (within the first five days) and during the initial phase of recovery (within the first three months after discharge). Postural stability can be attained by rehabilitation. Thus, a proper physiotherapy rehabilitation program can improve the quality of life.

## Introduction

The articulation of the femur’s head with the pelvic acetabulum occurs at the hip joint. Diarthrodial joints have three degrees of freedom: medial/lateral rotation in the transverse plane, abduction/adduction in the frontal plane, and flexion/extension in the sagittal plane. These two segments come together to form the joint [1]. Hip joint capsular ligaments (iliofemoral, ischiofemoral, and pubofemoral) are important for functional movement and joint stability [2]. Hemiarthroplasty has its own risks and complications [3]. In hip arthroplasty, deteriorated hip joint components are removed during surgery and replaced with prosthetic hip joints [4]. Reduced discomfort, more mobility, improved hip joint function, and an overall improvement in patient quality of life are the goals of hip replacement surgery. Technological advances have boosted the development of joint replacement. Both hips that are cemented and those that are not can offer long-term fixation. The use of large-bore bearings, which offer a broader range of motion while also enhancing stability and minimizing wear, has been made possible by better materials and design [5]. Hip replacement surgery is most commonly used in elderly patients, because of conditions like rheumatoid arthritis, osteoarthritis, and avascular necrosis of the femoral head caused by the fracture of the hip.

Physical rehabilitation plays a very important role in rehabilitation and attaining daily activities. After receiving a total hip replacement, patients benefit from rehabilitation in terms of pain, range of motion, weight-bearing, balance, hip strength, and gait velocity [6]. Technology has made rehabilitation much easier. We can have virtual rehabilitation for patients who cannot come to the clinic on a daily basis or for patients who are suffering from other musculoskeletal disorders [7]. Preoperative management is significant for attaining reliable results in conventional hip arthroplasty. Based on a comprehensive assessment of the clinical and radiological data, planning guides the surgeon in conceptualizing the operation [8]. This case report focuses on the rehabilitation of a patient who was operated on twice for total hip replacement. Rehabilitation protocol was provided for a period of 12 weeks, and there was an improvement in the Numerical Pain Rating Scale, Harris hip score, and lower extremity functional scale post-rehabilitation.

## Case presentation

Patient information

A 56-year-old man had a road traffic accident in 2020 in Chandrapur, the patient was taken to the hospital and after consulting with the orthopedic surgeon, an X-ray was done that revealed the acetabular fracture and fracture of the neck of the femur of the left side, the surgeon advised the total hip arthroplasty (THA) of the left side. The patient was operated on for the same and he was apparently alight in terms of pain and range of motion post one year after surgery.

**Figure 1:**
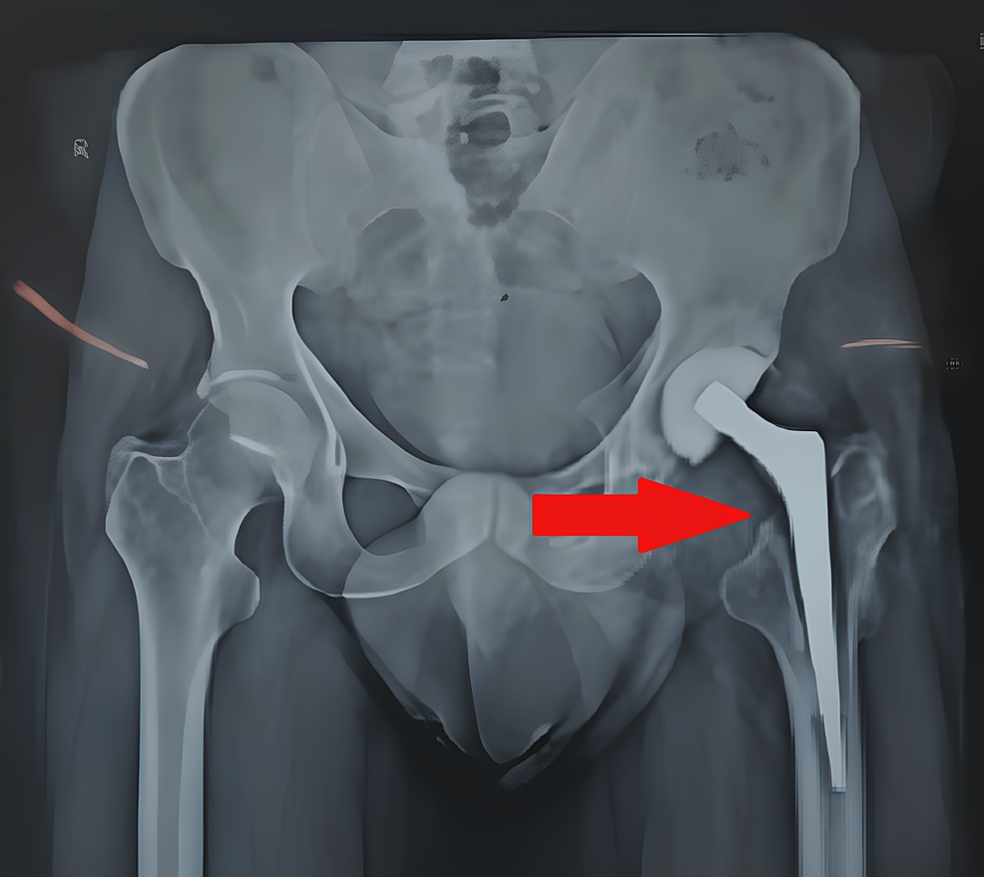
Postoperative X-ray after cemented implant (August 11, 2021)

**Figure 2:**
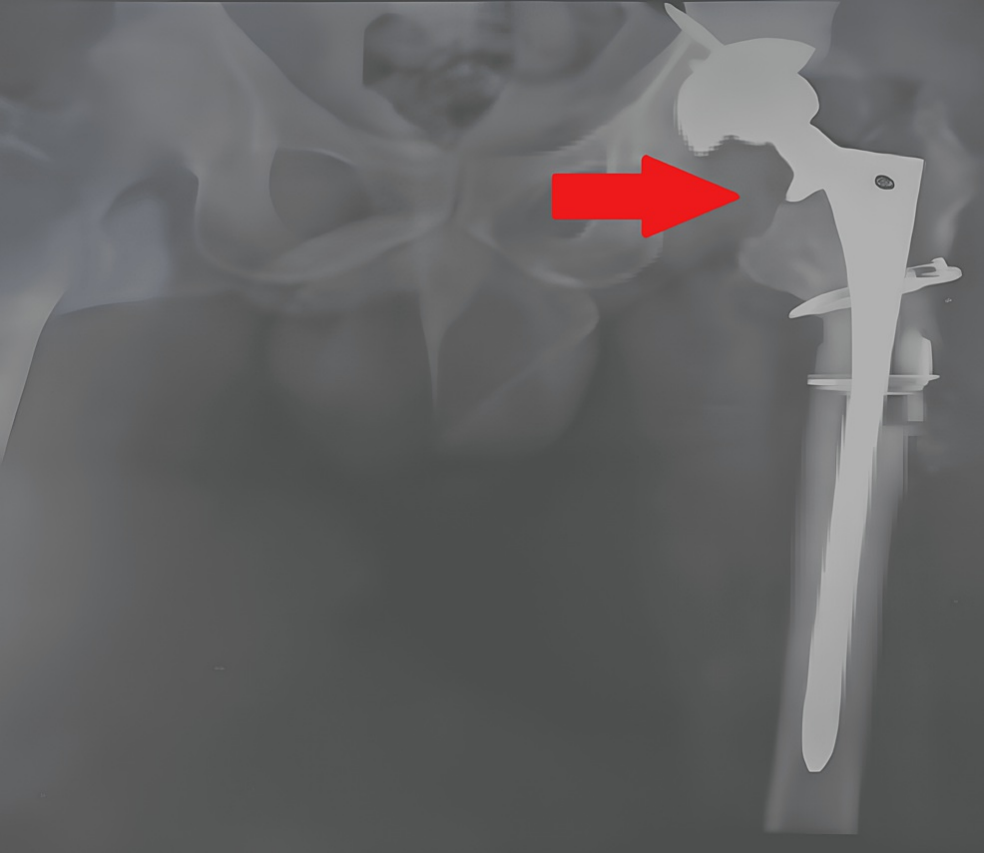
Postoperative X-ray after final metal implant surgery (September 27, 2022)

After a year of surgery in November 2021, the patient came to our tertiary care center with complaints of pain in the left hip. After consulting with the orthopedic surgeon, an X-ray scan (Figure 1) was done that revealed the infection in the implanted hip. There was a gradual onset of symptoms when he started complaining about pain in the limb and difficulty in maintaining balance or bearing weight on the left limb. The patient came with a complaint of pain which was insidious and gradually increasing and inability to walk. Removal of the implant was planned to decrease the infection of the left limb and temporary cement implanting was done. In August 2022, the patient was again admitted to our center for the revision of THA cemented of the left limb, and the surgery was performed on September 27, 2022 (Figure 2) for debridement and realignment of the implant of the left side.

Clinical findings

On inspection: The patient was seen in a supine position with both the lower extremities resting on the plinth. The right limb was normal and was able to perform all the movements actively while, the left limb was not able to perform flexion, extension, abduction, and adduction. The left knee was not able to flex. During the process of palpation, the patient exhibited a willingness to allow the therapist to touch. However, it was during the palpation that the therapist was able to confirm their findings. The muscles were intact and contracted throughout. On examination of the left hip: no swelling was present, but flexion deformity was observed. The hip range of motion was restricted and painful. The range of motion in both upper limbs was complete and functional at the shoulder, elbow, wrist, and hand joints (complete and pain-free). The implant used was the cemented implant for the left side. The pain gradually increased, restricting the range of motion and inability to walk. The pain was insidious and occurred mainly due to the infection caused by the implant. Pain on the numerical pain rating scale was 8/10. For both upper limbs, soft tissue tension in the muscles and ligaments around the wrist, elbow, and shoulder joints was nil. In the gluteus and thigh muscles of the left lower limb, soft tissue tension was observed. The hip is rigid, making it difficult to move or lift the leg. The total range of motion is constrained by hip stiffness.

Rehabilitation

**Figure 3:**
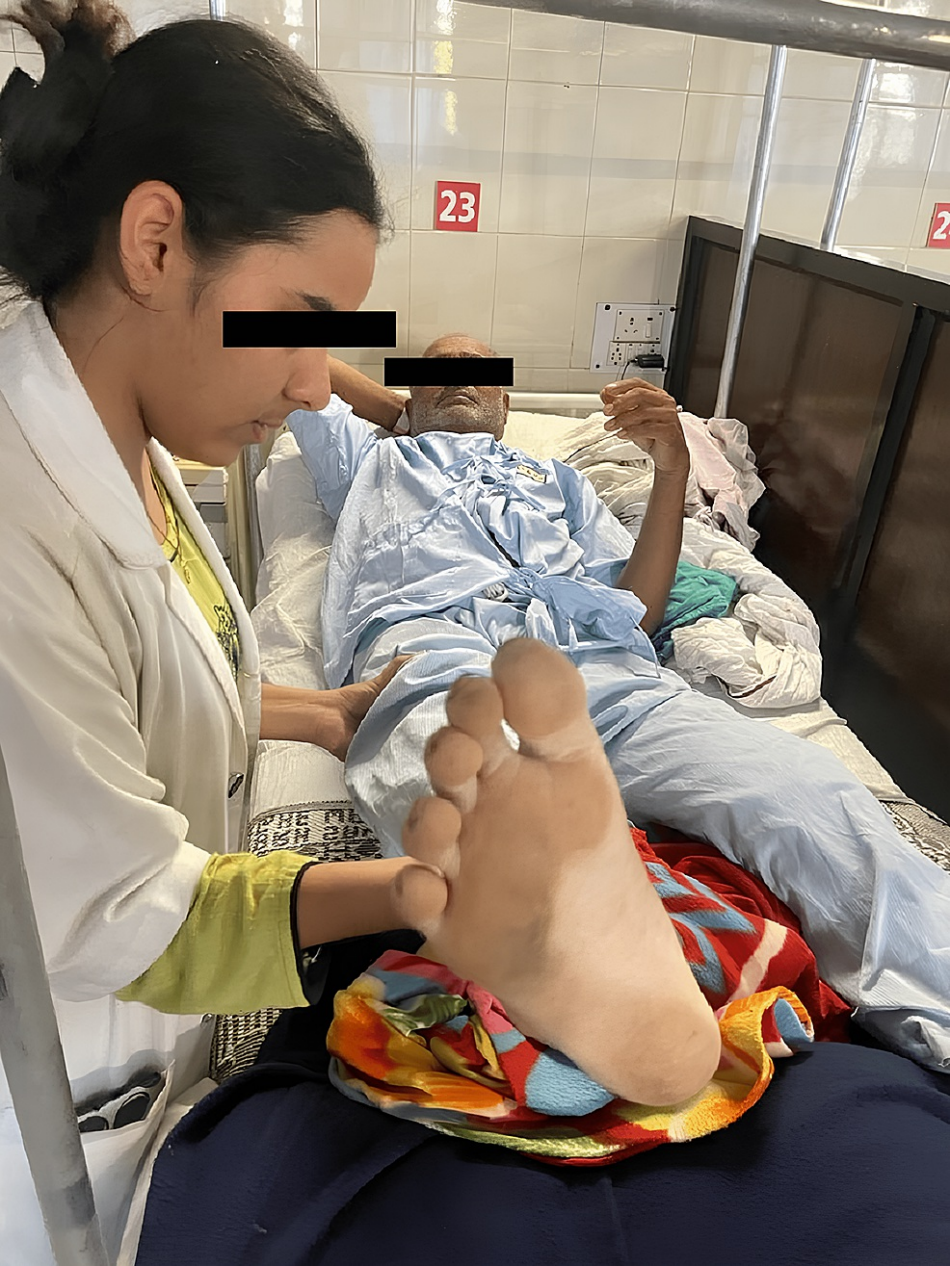
Phase II: Performing straight leg raises (with assistance).

The rehabilitation program is provided in Table 1 and Figure 3.

**Table 1:** Rehabilitation plan for the patient LE: lower extremity; ROM: range of motion

Sr no.	Goals	Intervention	Treatment Regimen
Phase I (day 1-2 weeks)			
1	Patient education	To educate the patient and his family about the patient’s condition, precautions to be taken, complications to be avoided, and how physiotherapy is important for recovery.	Counseling was done on the effects and importance of exercise, ambulation, and position.
2	To prevent secondary pulmonary and circulatory complications	Breathing exercises, use of spirometer, ankle pumps, on affected leg: static glutes, static quads, static hams, heel slides, active range of motion for unaffected side, hip abduction in gravity eliminated position	10 reps x 1 set each
3	Positioning	A pillow should be placed between the legs to avoid adduction and internal rotation of the hip. Avoid knee and hip flexion for the first 2 weeks.	It should be done in lying and long sitting. Positioning should be done every two hours.
4	To reduce pain and swelling	Cryotherapy	10-15 minutes, two times a day
Phase II (week 2-week 6)			
1	To regain the strength of the lower limb muscles after surgery	ROM: continue with all phase I exercises. Strengthening: sitting position: maintaining hip flexion. Standing dip flexion/abduction/adduction near the end of this period, progress to straight leg raises (Figure 3), and hip abduction, adduction, and extension against gravity. Knee ROM exercise bridging, progress to closed chain exercises (mini squats, step-ups, mini lunges) by the end of this phase.	Same as phase I. 15 minutes, twice a day. 10 reps x 1 set with 5 seconds of hold for 3 weeks followed by 10 seconds hold for 4 weeks and later on, 15 seconds hold for 5 weeks. 5 reps x 1 set
2	Initiate proprioceptive training	Weight shifting: Pelvic tilts on Swiss ball, single leg stance, standing on Bosu ball, squatting on Bosu ball	10 reps x 1 set and 5 reps x 1 set
3	To normalize all functional mobility	Gait training with appropriate device emphasizing normal gait pattern. Stair training with appropriate devices.	Two rounds of 15 m hallway initiated in the third week.
Phase III (6-12 weeks)			
1	To re-establish healthy LE strength, particularly healthy quadriceps activity. Returning to the fundamental functional tasks	ROM: continue with phase I and II exercises, strengthening: continue with phase II exercises, adding resistance as tolerated. Proprioception: Progress to gentle agility exercises, tandem walking, sidestepping, backward walking	10 reps x 1 set 10 minutes, twice a day starting from 7 weeks of rehabilitation
Phase IV (12 weeks and beyond)			
1	To maintain strength development in order to maximize functional results	ROM: Continue ROM exercises and stretching exercises as needed strengthening: continue with all strengthening exercises increasing the resistance, and decreasing repetitions. Proprioception: continue with all phase III exercises, increasing difficulty as tolerated.	10 reps x 1 set of all the exercises

Follow-up and outcomes

A carefully thought-out physical therapy interventional procedure was started. For 4 weeks, a follow-up was done once every week. Post-discharge, regular follow-up was taken via telerehabilitation. Tables 2-3 depict the outcome measure score.

**Table 2:** Outcome measures and score

Outcome Measure	On Admission	At Discharge
Numerical Pain Rating Scale	8/10	4/10
Harris hip score	22/100	70/100
Lower extremity functional scale	7/80	38/80

**Table 3:** Range of motion of lower limb (in degrees)

Joint	Movement	On admission	At discharge	On admission	At discharge
		LEFT	LEFT	RIGHT	RIGHT
Hip	Flexion	0° - 10°	0°- 80°	0°- 130°	0° - 130°
	Extension	0°	0° - 10°	0° - 30°	0° - 30°
Knee	Flexion	0° - 10°	0°- 60°	0° - 120°	0° - 120°
	Extension	0°- 10°	0° - 60°	0°- 120°	0° - 120°
Ankle	Plantarflexion	0° - 30°	0°- 35°	0° - 40°	0° - 40°
	Dorsiflexion	0° - 15°	0° - 20°	0° - 25°	0° - 25°

## Discussion

Here we discuss a case of a 56-year-old male who underwent revision total hip replacement surgery and was referred for physiotherapy. The primary goal of physiotherapy management was the prevention of secondary complications, promoting the strength and ROM of the lower limb, and promoting weight-bearing as early as possible.

Smith et al. in a study demonstrate that it is safe and successful, with a focus on early weight-bearing and ambulation [8-9]. It has been demonstrated that early intervention reduces mortality and morbidity, and promotes functional recovery in senior hip fractures. Limiting a patient’s ability to bear weight after surgery makes it more difficult for them to recuperate since they will need to use walking aids for a longer amount of time and stay in an extended care facility. By requiring quick completion, early weight-bearing will encourage effective regeneration while maintaining modest levels of complications in distal femoral peri-prosthetic fractures [9]. Schmitt-Sody and Valle placed a strong emphasis on pain management, proper positioning, reducing edema, preventing pressure ulcers, and prevention of pneumonia. In the first stage of rehabilitation, the patient was spoken to and taught how to sit and stand while carrying a partial weight. Now, the patient needs to transition out of bed [10]. In the research of Paterno et al. and Paterno and Archdeacon, phase I workouts were mostly focused on hip and knee joint mobility exercises, non-weight-bearing strengthening, and weight-bearing progression while walking [10-12]. The patient had to exhibit a 50% outpatient weight-bearing capability, strong quadriceps femoris muscle contraction, and good hip abductor muscle tension before moving on to phase II. Exercises in phase III focused on improving mobility, balance, preconception, and physical conditions while also strengthening the lower extremities and completing weight-bearing exercises [11-12].

## Conclusions

Physiotherapy rehabilitation plays an important role in the treatment plan of a patient with total hip replacement. Physiotherapy helps in early weight-bearing and making the patient independent. This case study demonstrates how rehabilitation can help a patient with a total hip replacement achieve better functional results. Exercises that improve range of motion, strength, gait, and balance are the main components of the treatment program. The patient’s functional abilities, mobility, and pain management were significantly improved. It also demonstrates the need for treatment plans to be specifically adapted to the needs and goals of each patient. For patients with total hip replacements, physical therapy rehabilitation can offer a secure and efficient method for achieving optimal healing and regaining their quality of life.
